# Differentially expressed microRNAs in peripheral blood mononuclear cells of non‐segmental vitiligo and their clinical significance

**DOI:** 10.1002/jcla.23648

**Published:** 2020-11-10

**Authors:** Zhibin Zhang, Xinyue Yang, Ougen Liu, Xianwei Cao, Jianbo Tong, Ting Xie, Jie Zhang, Yating Peng

**Affiliations:** ^1^ Department of Dermatology The Second Affiliated Hospital of Nanchang University Nanchang China; ^2^ Department of Dermatology The First Affiliated Hospital of Nanchang University Nanchang China

**Keywords:** bioinformatics analysis, microRNAs, non‐segmental vitiligo, peripheral blood mononuclear cells, RNA sequencing

## Abstract

**Background:**

Vitiligo is a frequent acquired depigmentation skin disease due to a loss of melanocytes. This study sought to characterize the expression pattern of microRNA (miRNA) in the peripheral blood mononuclear cells (PBMCs) of non‐segmental vitiligo (NSV) patients. We also screened for molecular markers that can be used to evaluate the clinical stages of NSV.

**Methods:**

The miRNA expression profile in the PBMCs of four patients with progressive NSV and four healthy controls was determined using high‐throughput RNA sequencing. The divergently expressed miRNA was verified via qRT‐PCR in 26 progression, 26 stable NSV, and 26 healthy controls.

**Results:**

Our findings posited that 323 miRNAs were differentially expressed in the PBMCs of NSV patients. The top 10 up‐regulated miRNAs in patients were hsa‐miR‐335‐5p, hsa‐miR‐20a‐5p, hsa‐miR‐514a‐3p, hsa‐miR‐144‐5p, hsa‐miR‐450b‐5p, hsa‐miR‐369‐3p, hsa‐miR‐101‐3p, hsa‐miR‐142‐5p, hsa‐miR‐19b‐3p, and hsa‐miR‐340‐5p. The top 10 down‐regulated miRNAs in patients were hsa‐miR‐4443, hsa‐miR‐1248, hsa‐miR‐6859‐3p, hsa‐miR‐668‐3p, hsa‐miR‐7704, hsa‐miR‐323a‐5p, hsa‐miR‐1237‐3p, hsa‐miR‐3127‐3p, hsa‐miR‐6735‐3p, and hsa‐miR‐127‐3p. The expressions of hsa‐miR‐20a‐5p in PBMCs of progressive and stable NSV were remarkably elevated relative to the healthy controls. In the characteristics curve analysis of hsa‐miR‐20a‐5p for differentiating progressive and stable NSV from normal subjects in PBMCs, the area under curve (AUC) was 0.92 and 0.81. Compared with patients in stable NSV, the hsa‐miR‐20a‐5p was markedly increased in PBMCs of progressive NSV patients, and the AUC was 0.81.

**Conclusion:**

Our results showed that divergently expressed miRNAs contribute to the pathogenesis of NSV and that hsa‐miR‐20a‐5p can be applied as a biosignature for stage assessment in PBMCs of patients with NSV.

## Graphical abstract

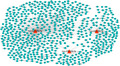

## 1 INTRODUCTION

Vitiligo constitutes a frequent acquired depigmentation skin disease due to a loss of melanocytes. It occurs worldwide with an estimated prevalence of 0.2% in community‐hinged studies and 1.8% in hospital‐centered studies.^1^ It is a complex disease caused by the interaction of genetic, immune abnormalities, and psycho‐psychological factors.^2^ The clinical manifestations are depigmentation spots of different sizes and shapes in the skin and mucosa. Vitiligo is easily diagnosed but difficult to treat. It affects the physical beauty and torture to patients, thereby reducing their quality of life.^3^

The origin of the degeneration of epidermal melanocytes is intricate and remains unclear. Diverse theories have been proposed: One of the theories suggests that melanocytes destroy themselves through autoimmune mechanism (mainly cellular immunity).^4^ Peripheral blood mononuclear cells (PBMCs) are composed of lymphocytes (T, B lymphocytes), NK cells, monocytes, and dendritic cells in the blood circulation. These cells form an important part of the immune system because many immune cells migrate from the blood circulation to the skin. In addition, PBMCs in patients with vitiligo over‐produce proinflammatory cytokines consisting of interleukin (IL)‐1b, IL‐8, IL‐6, and the tumor necrosis factor (TNF)‐α.^5^ The cytokines infiltrate around the vitiligo lesions, indicating that PBMCs are closely related to the pathogenesis of vitiligo. However, its specific pathogenesis is still unclear.

In recent years, genome‐wide microRNA (miRNA) studies have been extensively used to elucidate the genetic and immune‐correlated pathogenesis of vitiligo. miRNAs constitute a kind of non‐coding RNA (ncRNA) approximately 23 nucleotides long. They are present in eukaryotic organisms, and control post‐transcriptional gene expression by repressing translation or enhancing the degeneration of mRNA in the cytoplasm.^6^ With the discovery of gene chips and RNA sequencing technology, researchers have realized the pivotal function of miRNAs in the modulation of human gene expression. It has been shown that miRNAs were associated with complex diseases, and may become biosignatures for the diagnosis and therapy of complex diseases.^7^ So far, reports have revealed that miRNAs were abnormally expressed in skin tissue, serum, and PBMCs of vitiligo. For example, Shi and coworkers have found that miR‐16, miR‐19b, and miR‐720 were effective serological markers to differentiate NSV from healthy peoples.^8^ However, studies on miRNA expression profiles and functional analysis in PBMCs of patients with NSV at different stages have not been reported.

The primary purpose of this research was to analyze the differential miRNA expression profiles between the PBMCs of patients with progressive NSV and healthy individuals. The potential functions of different miRNAs in the pathogenesis of vitiligo were analyzed to provide a theoretical foundation for further research.

## 2 MATERIALS AND METHODS

### 2.1 Subject information and sample collection

Fifty‐six patients, as well as 30 age‐ and sex‐corresponding healthy control individuals, were recruited in this study at our hospital from July 2019 to May 2020. Based on clinical symptoms, they were divided into progressive NSV (among four cases were used for RNA‐seq) and stable NSV. The progressive vitiligo patients had enlarged lesions with new lesions forming within 6 months and with a Vitiligo European Task Force (VETF) transmission score of +1 to +5. The stable vitiligo patients had no increase in size within 6 months and had a VETF score of −5 to 0 which is considered stable vitiligo.^9^, ^10^ The protocols used in this study were ratified by the ethics review committee. All the subjects signed a written informed consent. All subjects were not accompanied by other organic, autoimmune, and infectious diseases. The patients were not systematically treated with glucocorticoids, immunosuppressants, photosensitizers, and ultraviolet rays within 1 month.

### 2.2 PBMC isolation and RNA extraction

Peripheral venous blood (5 mL) was collected from patients with progressive NSV, stable NSV, and healthy controls. The PBMCs were isolated from the human peripheral blood lymphocyte using the density‐gradient centrifugation method and maintained at −80°C awaiting RNA purification. We employed the TRIzol Kit (Life Technologies) in purifying the total RNA from the PBMCs as outlined in the protocol of the manufacturer. The RNA quality was inspected by a ND‐1000 NanoDrop (Thermo Fisher) and Agilent 2200 Bioanalyzer (Agilent Technologies).

### 2.3 RNA sequencing and bioinformatics analysis

A cDNA library of RNA samples was constructed by using VAHTSTM Total RNA‐seq (H/M/R) as per the manufacturer's protocol. The cDNA library was paired and sequenced on IIIumina HiSeqTM2500 at RiboBio Co. Ltd. We analyzed the miRNA expression clustering and the differential expression among samples. The differentially expressed miRNA between samples was picked based on difference multiple (|log2(Fold_Change)| ≥ 1) and the significance level (*P* < .05). Volcano plots were used to count the overall distribution of differential miRNAs (|log2(Fold_Change)| ≥ 1). Hierarchical clustering analysis (|log2 (Fold_Change)| ≥ 1) was employed to analyze the expression of divergently transcribed miRNAs under various experimental parameters. The significant enrichment analysis of parental genes of the divergently expressed miRNAs was carried out hinged on the GO and KEGG database.

### 2.4 Verification of candidate miRNAs

Reverse transcription of the RNA was accomplished using the reverse transcription kit (Takara) the total RNA to cDNA. The selected miRNAs (relatively plentiful, |log2(Fold_Change)| > 2 and *P* < .001) were verified via qRT‐PCR. The content of seven miRNAs (hsa‐miR‐127‐3p, hsa‐miR‐20a‐5p, hsa‐miR‐142‐5p, hsa‐miR‐340‐5p, hsa‐miR‐101‐3p, hsa‐miR‐335‐5p, and hsa‐miR‐668‐3p) was estimated by qRT‐PCR on the ABI Prism 7900 system (Applied Biosystems). U6 was employed as an internal reference for normalization. All miRNA primers were synthesized by RiboBio Co. Ltd. The 2−△△ct method was used to quantitatively analyze the results.

### 2.5 Structure of miRNA‐mRNA network

miRNA target genes were predicted by miRTarBase, miRDB, TargetScan, and miRWalk. The final predicted target genes were acquired by three software (any three of the four mentioned above). Cytoscape 3.6.1 was used to construct miRNA and predict target gene network.

### 2.6 Statistical analysis

All data were counted by GraphPad Prism version 7.0. We used Student's *t* test to analyze normally distributed measurement data between two arms. Comparisons among multiple study arms were inspected by one‐way ANOVA. The NSV screening by hsa‐miR‐20a‐5p level was estimated by receiver operating characteristic (ROC) curve assessments in PBMCs of NSV and healthy controls. The specificity and sensitivity of predictive power were assessed via the area under curve (AUC). *P* < .05 signified statistical significance. Correlation evaluation was conducted via the Pearson correlation assessment.

## 3 RESULTS

### 3.1 Expression profile of miRNAs in PBMCs of NSV

**FIGURE 1:**
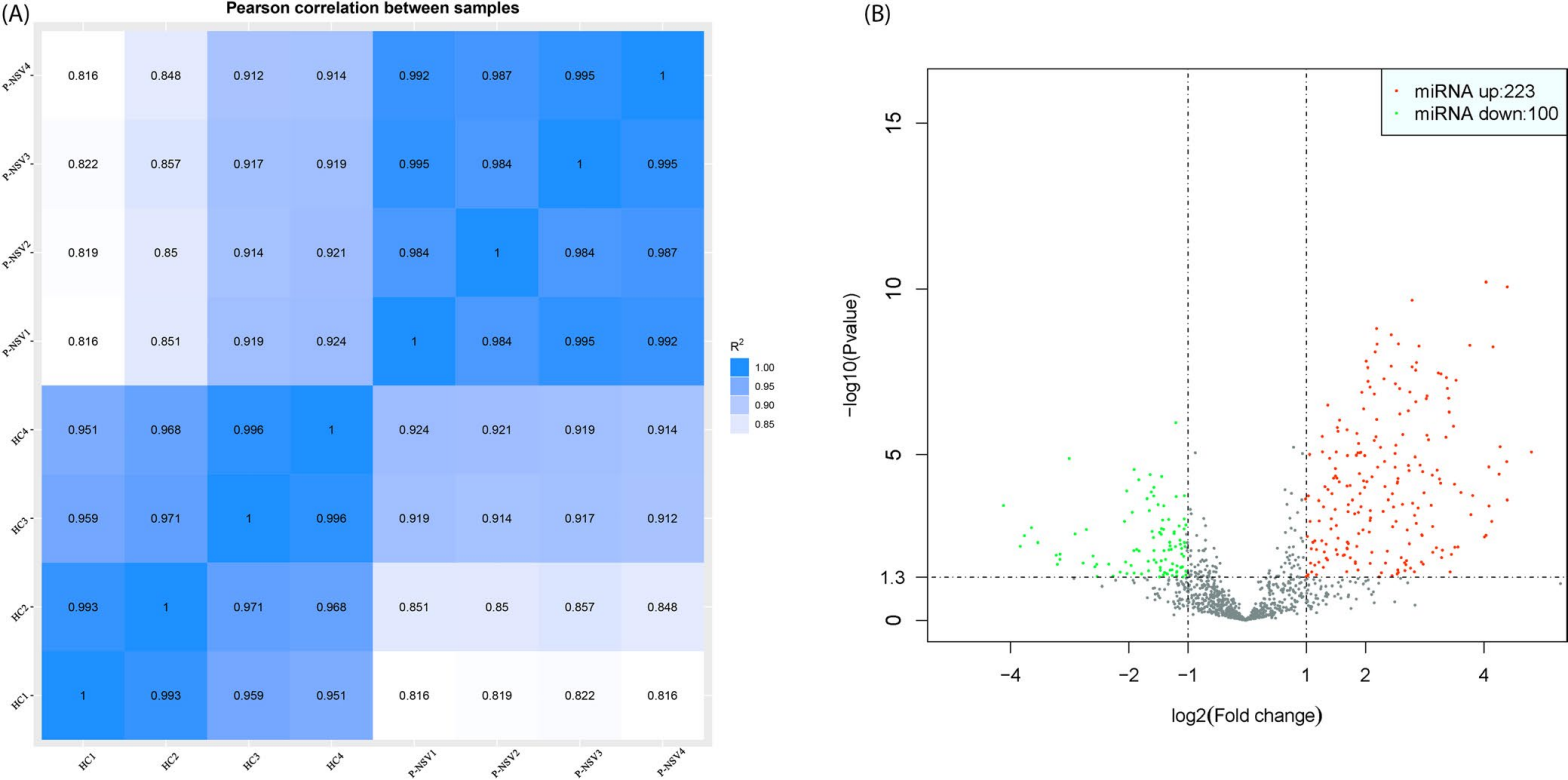
Expression pattern of miRNAs in progressive non‐segmental vitiligo (P‐NSV) and healthy controls (HC) as determined by RNA‐seq. A, The scatter plot shows the correlation analysis between all samples. The greater the correlation coefficient and the darker the color (blue squares, pale blue squares, and white squares), the higher the relationships of samples. B, Volcano diagram shows divergently expressed miRNAs between the two arms. Red dot represents up‐regulation, and green dot represents down‐regulation

**FIGURE 2:**
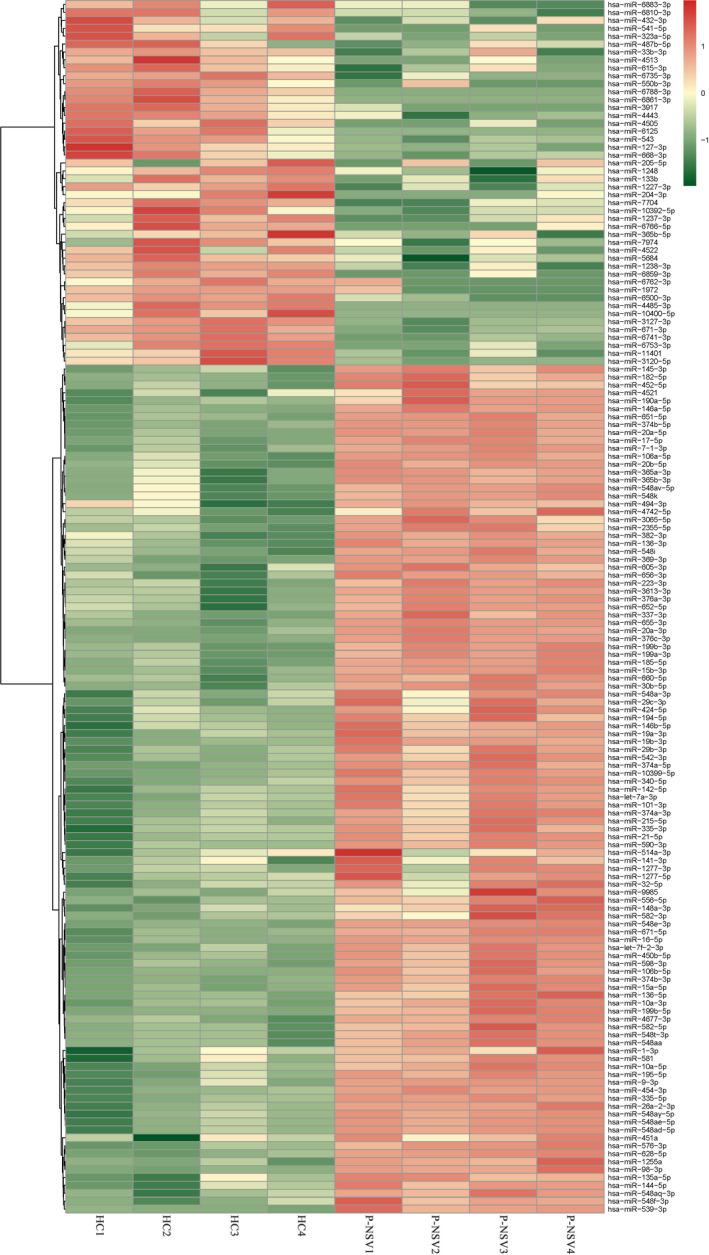
Expression profile of miRNAs in the two arms. Hierarchically grouped heat map present miRNAs expression profile, with red represent up‐regulation and green represents down‐regulation

**FIGURE 3:**
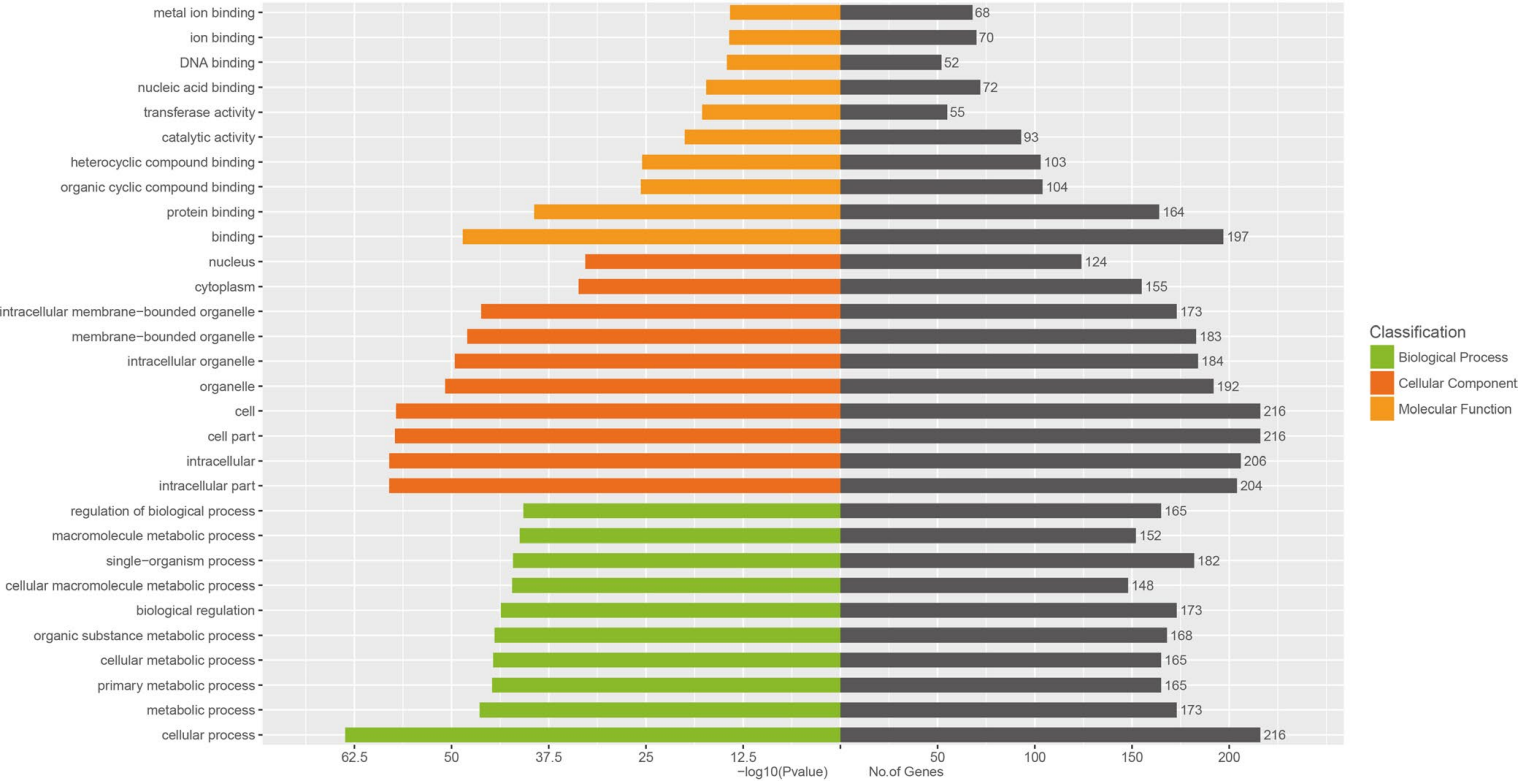
The top 10 GO enrichment terms. GO assessment was conducted on the parental genes of miRNAs with different expression levels (|log2Fold_Change| ≥ 1, *P* < .05). The significant threshold is *P* < .05, which has obtained statistically significant biological functions and pathways. GO enrichment analysis showed that changes in biological processes of differential miRNAs mainly occurred in cellular, metabolic, primary metabolic, and cellular metabolic

**FIGURE 4:**
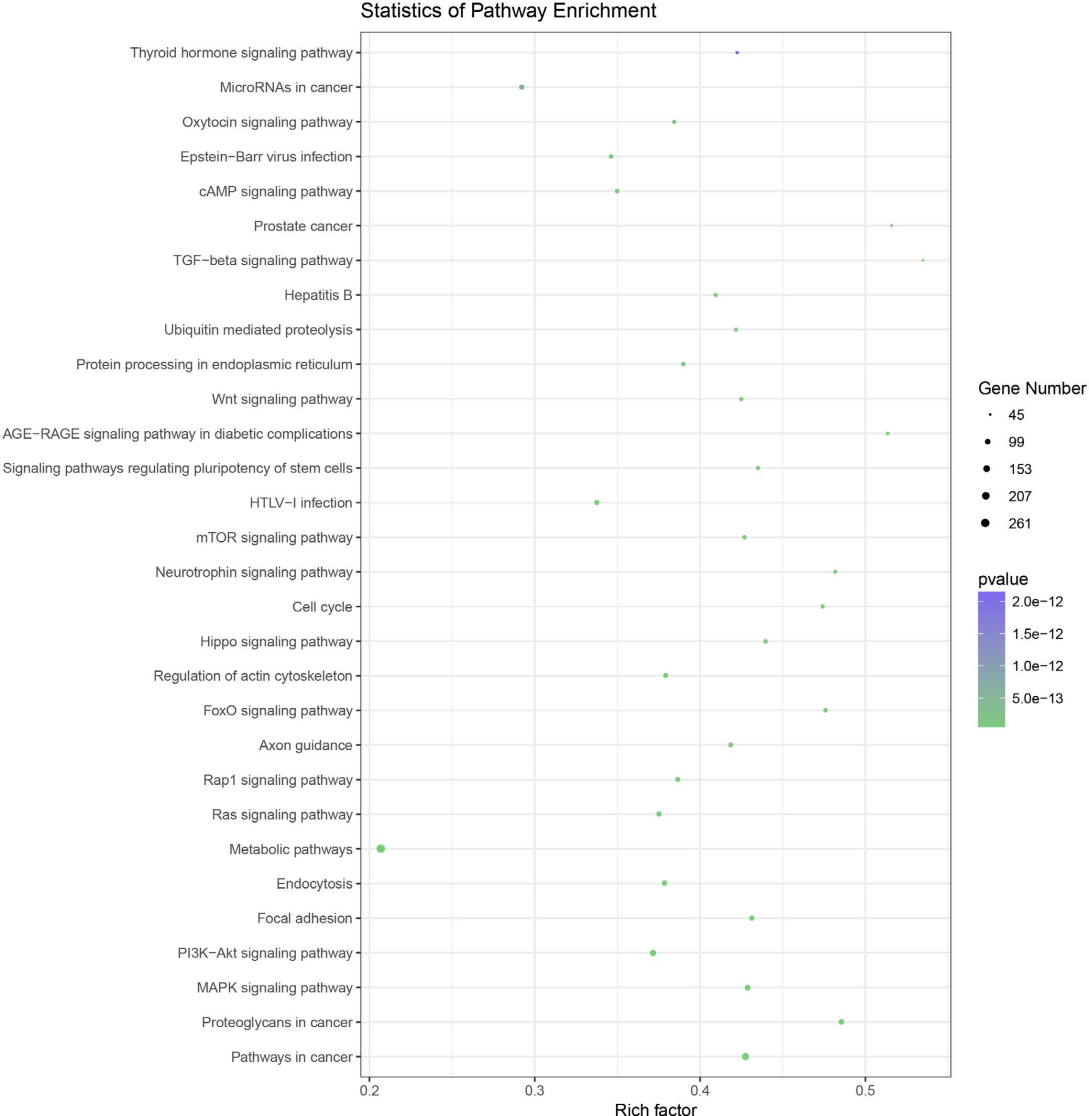
The top 30 KEGG pathways are enriched in terms. The KEGG pathway assessment revealed that TGF‐beta, mTOR, focal adhesion, and PI3K‐Akt signaling cascade were significantly related to divergently expressed miRNAs in NSV

The sequencing data were normalized and compared between the two arms. The Pearson correlation coefficient (*r*) between progressive NSV (P‐NSV) and healthy controls (HC) is shown in Figure 1A. High‐throughput RNA sequencing was performed on RNA extracted from PBMCs of four progressive NSV patients and four healthy controls. The findings disclosed that there were 323 miRNAs remarkable differences in the progressive NSV arm relative to the healthy control arm. The volcano plots disclosed the divergently expressed miRNAs in the progressive NSV and the healthy control (Figure 1B). Collectively, 323 miRNAs showed differential expressions in progressive NSV relative to the healthy control. The top 10 up‐ and down‐regulated miRNAs were summarized in Table 1. Hierarchical grouping assessment was conducted on divergently expressed miRNAs (Figure 2). Red indicated relatively high expression miRNAs, and green indicated relatively low expression miRNAs. GO enrichment analysis showed that changes in biological processes of differential miRNAs mainly occurred in cellular, cellular metabolic and primary metabolic, and cellular metabolic functions. Changes in molecular metabolic functions were mainly focused on binding, protein binding, and organic cyclic compound binding. The cell composition analysis showed differences in how most miRNAs are involved in the intracellular, cell, and organelle composition (Figure 3). KEGG pathway assessment showed that differential mRNAs are mainly enriched in TGF‐beta, mTOR, focal adhesion, and the PI3K‐Akt signaling pathway (Figure 4).

**TABLE 1:** The top 10 up‐regulated and down‐regulated miRNAs in the PBMCs of NSV patients

MicroRNA	Log2(Fold_change)	Up/Down	*P*‐value
hsa‐miR‐335‐5p	4.423433796	Up	.0000
hsa‐miR‐20a‐5p	4.064873062	Up	.0000
hsa‐miR‐514a‐3p	3.537779439	Up	.0006
hsa‐miR‐144‐5p	3.532592122	Up	.0000
hsa‐miR‐450b‐5p	3.412136094	Up	.0000
hsa‐miR‐369‐3p	3.395145218	Up	.0000
hsa‐miR‐101‐3p	3.387655138	Up	.0000
hsa‐miR‐142‐5p	3.304522931	Up	.0000
hsa‐miR‐19b‐3p	3.258739228	Up	.0000
hsa‐miR‐340‐5p	3.055838262	Up	.0000
hsa‐miR‐4443	−3.975703094	Down	.0000
hsa‐miR‐1248	−3.042051153	Down	.0010
hsa‐miR‐6859‐3p	−3.021829745	Down	.0017
hsa‐miR‐668‐3p	−3.004760912	Down	.0001
hsa‐miR‐7704	−2.911161837	Down	.0005
hsa‐miR‐323a‐5p	−2.883165814	Down	.0007
hsa‐miR‐127‐3p	−2.880054908	Down	.0000
hsa‐miR‐1237‐3p	−2.880054908	Down	.003
hsa‐miR‐3127‐3p	−2.879118895	Down	.0002
hsa‐miR‐6735‐3p	−2.835809793	Down	.0007

### 3.2 Verification of the divergently expressed miRNAs

**FIGURE 5:**
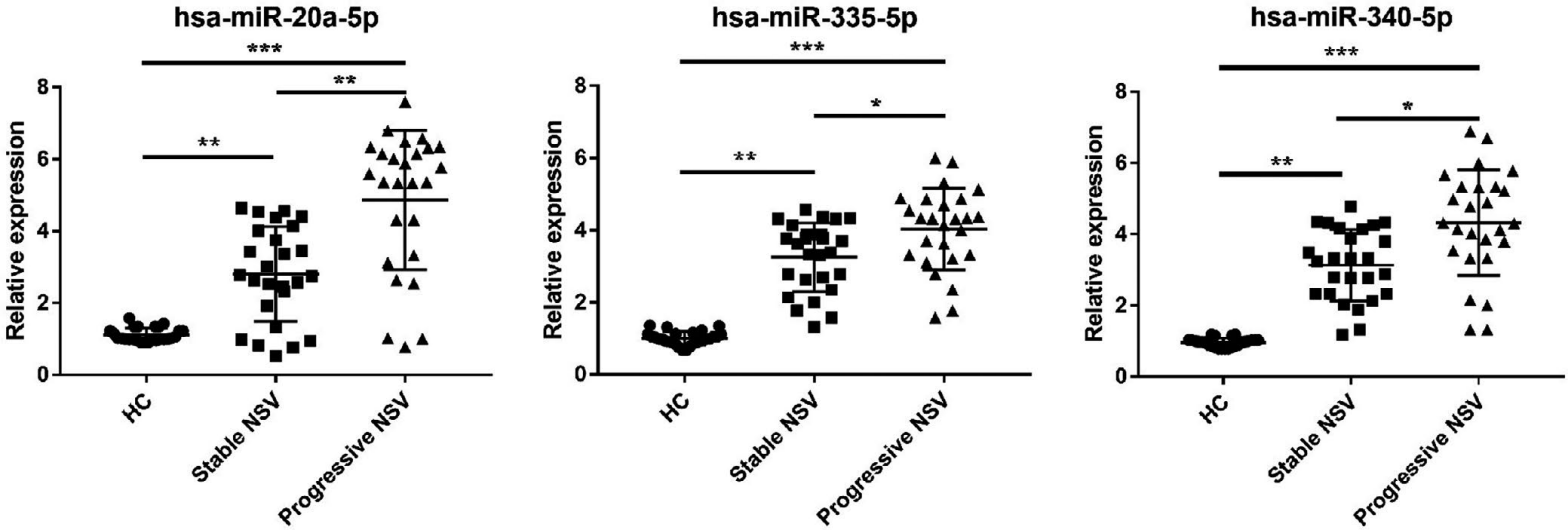
Verification of candidate miRNAs. The expression levels of the candidate miRNAs in 26 progression NSV, 26 stable NSV, and 26 healthy controls samples by qRT‐PCR (*P < .05, **P < .01, ***P < .001)

qRT‐PCR was employed to inspect the expression level of seven divergently expressed miRNAs (relatively plentiful, |log2(Fold_Change)| > 2 and *P* < .001) in the progressive NSV, stable NSV, and healthy individuals. Results showed that the expressions of hsa‐miR‐20a‐5p, hsa‐miR‐335‐5p, and hsa‐miR‐340‐5p in PBMCs of progressive and stable NSV were markedly higher relative to the control arm (*P* < .001). Relative to the stable phase NSV patients, hsa‐miR‐20a‐5p was increased in PBMC progressive NSV patients remarkably (Figure 5).

**FIGURE 6:**
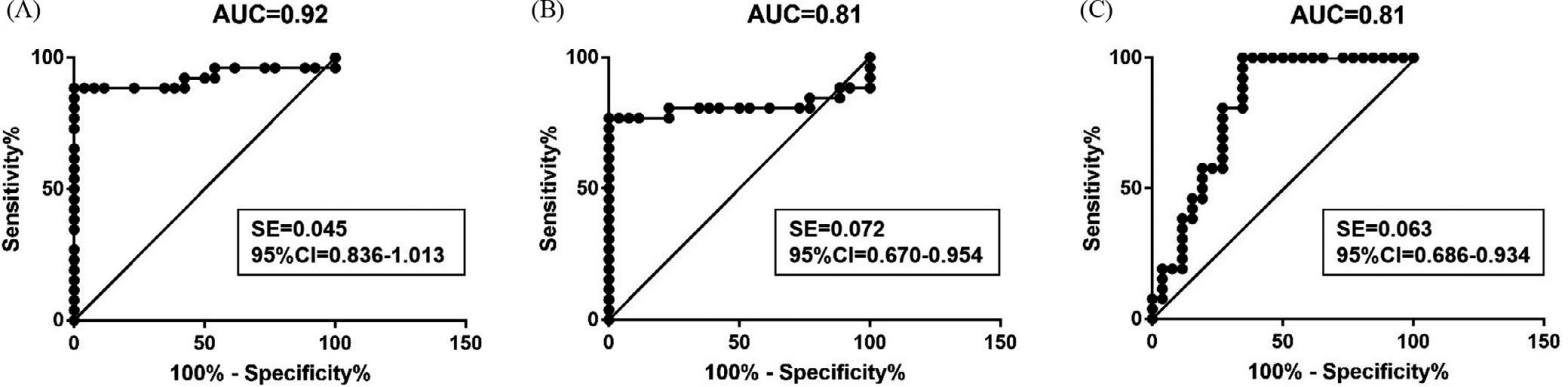
The receiver operating characteristic (ROC) curve of PBMCs hsa‐miR‐20a‐5p level. The ROC curve of hsa‐miR‐20a‐5p level based on true‐positive and false‐positive ratios in screening is shown. The area under curve (AUC) of hsa‐miR‐20a‐5p level was 0.92 (A, progressive NSV vs healthy controls), 0.81 (B, stable NSV vs healthy controls), and 0.81 (C, progressive NSV vs stable NSV

We then assessed the possibility of hsa‐miR‐20a‐5p as a prospective disease marker for NSV. ROC curve inspection unearthed that the PBMCs hsa‐miR‐20a‐5p level could serve as an effective biosignature for differentiating progressive and stable NSV patients from healthy individuals with the AUC of 0.92 and 0.81 (Figure 6). More importantly, the PBMC hsa‐miR‐20a‐5p level could serve as an effective marker for differentiating progressive NSV from stable NSV patients with the AUC of 0.81 (Figure 6).

### 3.3 miRNA‐mRNA network prediction

**FIGURE 7:**
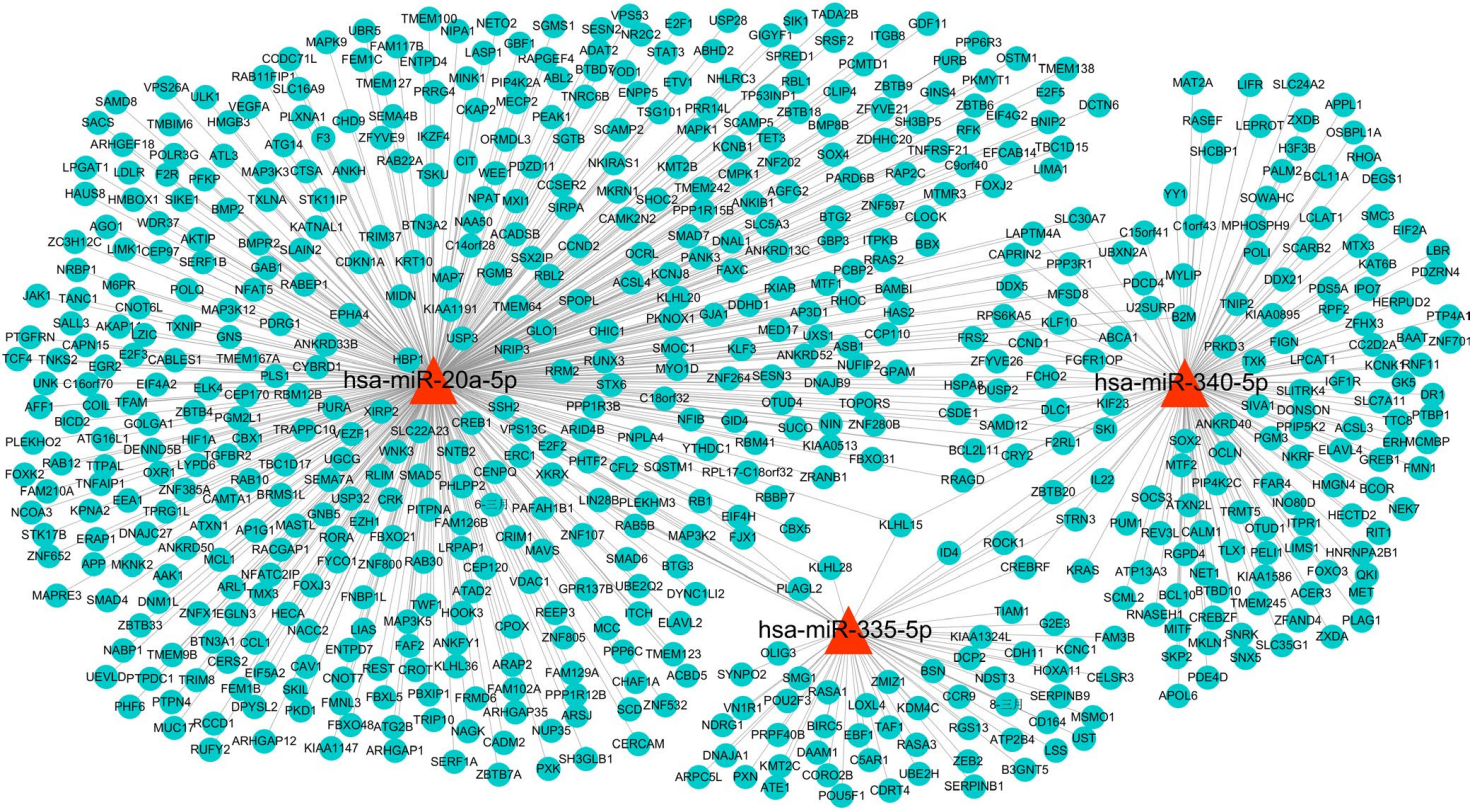
Structure of miRNA‐mRNA network. The miRNA‐mRNA network was constructed using miRNAs (hsa‐miR‐20a‐5p, hsa‐miR‐335‐5p, and hsa‐miR‐340‐5p). miRNAs (red triangles) and their targeted mRNAs (blue ellipses) were predicted using prediction algorithms (miRTarBase, miRDB, TargetScan, and miRWalk)

Three divergently expressed miRNAs (hsa‐miR‐20a‐5p, hsa‐miR‐340‐5p, and hsa‐miR‐335‐5p) and mRNA network were constructed in progressive NSV. Hsa‐miR‐20a‐5p contained 468 target genes, hsa‐miR‐335‐5p contained 65 target genes, while hsa‐miR‐340‐5p had 151 target genes. The relationship between miRNAs and target genes is indicated in Figure 7. The predicted target mRNA (blue ellipses) of three significantly differential miRNAs (red triangles) was drawn using Cytoscape3.6.1 (Figure 7).

## 4 DISCUSSION

Vitiligo is a common depigmented skin disease, characterized by localized or generalized leukoplakia at the skin and mucous membranes. Currently, vitiligo treatments focus on controlling the development of skin lesions and promoting the recovery of white spots.^2^ Vitiligo treatment is specific to each patient since the severity varies from one patient to the next. Evaluation of the vitiligo stage is extremely important for the selection of vitiligo treatment options and efficacy. However, there is currently no consistent method for clinical evaluation of vitiligo activity. Therefore, it is great significance to find indicators for assist evaluation of vitiligo activity.

miRNAs constitute endogenous non‐coding RNAs composed of approximately 23 nucleotides. They specifically bind to the 3′ untranslated region of the target mRNAs. They also suppress the expression of target mRNAs, initiate post‐transcriptional level regulation of target mRNAs, and participate in about 30% of human genome regulation.^6^ Several studies have shown that miRNA can be used as an evaluation indicator for diagnosis, staging, and severity of various diseases.^11^, ^12^ The miRNAs have been found to be closely related to the pathogenesis of vitiligo. Shi and coworker reported that miR‐25 increased in serum of vitiligo.^13^ Further research showed research has shown that miR‐25 directly inhibits the expression of microphthalmia‐associated transcription factor (MITF), which is the main modulator affecting melanocyte survival and function. It does this by combining with the 3′UTR region of MITF mRNA, thus promoting melanocyte apoptosis. More importantly, miR‐25 also represses the generation and secretion of stem cell factor, as well as the basic fibroblast growth factor in keratinocytes. This results in the weakening of the protective effect of paracrine on melanocytes under oxidative stress, resulting in depigmentation and development of vitiligo. Reports have disclosed that miR‐16, miR‐19b, and miR‐720 are effective markers that can be used to differentiate non‐segmental vitiligo from healthy controls.^8^ These studies provide theoretical evidence for the role of miRNAs in vitiligo.

Previous research evidence chronicled that multiple components in PBMCs participate in the pathogenesis of vitiligo.^14^ Analysis of divergently expressed miRNAs in PBMCs of patients with NSV exhibited that miR‐224‐3p and miR‐4712‐3p were upmodulated and miR‐3940‐5p expression was down‐regulated relative to the healthy controls. Further research disclosed that thymosin ɑ1 may regulate the immune response of NSV through miRNAs, leading to the occurrence and development of NSV.^15^ However, miRNA chip detection is relatively narrow and cannot detect unknown miRNA. In addition, the relationship between miRNAs in PBMCs and NSV disease activity has not been reported.

In this study, high‐throughput RNA sequencing was employed to analyze the expression pattern of miRNA between progressive NSV and healthy people. We additionally studied the relationships between abnormal expression of miRNAs and NSV activities. We found a large number of divergently expressed miRNAs, of which 223 miRNAs were markedly upmodulated and 100 downmodulated. Previous studies have shown that the miRNAs family is a considerable part of the gene expression regulatory network. Determination of miRNAs target genes is crucial for comprehending the biological roles of miRNAs. The functional analysis of target genes is helpful to conjecture the possible function of miRNAs.^16^ A single miRNA can act on many target genes to regulate gene expression, while multiple miRNAs can also adjust certain target genes. We established that divergently expressed miRNAs participated in the modulation of 43 144 target genes based on GO analysis. They are mainly involved in combined, cell periphery, and single biological processes. The KEGG pathway analysis implied that focal adhesion,^17^ TGF‐beta,^18^ mTOR,^19^ and PI3K‐Akt signaling pathway were markedly contacted for divergently expressed (*P* < .05) miRNAs in NSV.^20^ Therefore, abnormal expression of miRNAs might be participating and modulating the development of NSV.

Subsequently, we selected seven miRNAs (relatively plentiful, |log2(Fold_Change)| > 2 and *P* < .001) for further evaluation. The results showed that the expression of hsa‐miR‐20a‐5p, hsa‐miR‐335‐5p, and hsa‐miR‐340‐5p in PBMCs of progressive and stable NSV was significantly higher than healthy controls. It is speculated that the divergently expressed miRNAs might serve a significant role in the pathogenesis of vitiligo. Further analysis revealed that hsa‐miR‐20a‐5p was significantly increased in PBMCs of progressive NSV patients relative to the patients in stable phase NSV. This implies that hsa‐miR‐20a‐5p was associated with vitiligo activity. The ROC curve assessment disclosed that the PBMC hsa‐miR‐20a‐5p level could serve as an effective biosignature for differentiating the different stages of NSV. Oxidative stress, an important initiating factor, can participate in initiating specific T cell immune responses against melanocytes by activating innate immune responses, causing melanocyte damage.^21^ Dong et al^22^ found that hsa‐miR‐340‐5p can inhibit hypoxia/reoxygenation‐triggered cardiomyocyte apoptosis, as well as oxidative stress by adjusting the Act1/NF‐κB pathway. In addition, Liu et al^23^ detailed that hsa‐miR‐335‐5p can targetedly inhibit sKlotho and promote endothelial cell oxidative stress‐mediated aging. Although the specific functional mechanism of hsa‐miR‐335‐5p and hsa‐miR‐340‐5p in vitiligo is still unknown, the increased expression of hsa‐miR‐335‐5p and hsa‐miR‐340‐5p may serve a regulatory function in vitiligo melanocytes under oxidative stress. Further research should be conducted to define the detailed function of hsa‐miR‐335‐5p and hsa‐miR‐340‐5p in vitiligo. Various studies have shown that hsa‐miR‐340 serves a critical role in modulating UVB‐triggered dendrite generation and melanosome transport.^24^ Therefore, we speculate that hsa‐miR‐340‐5p serves a pivotal role in the transfer of vitiligo melanin.

Network analysis links different miRNAs with target genes and related biological functions. This helps to better understand the role of deregulated miRNAs. It is predicted from biological information that hsa‐miR‐20a‐5p targets more than 468 genes. Previous studies have chronicled that the expression of F2RL1 mRNA and protein in CD8 + T cells in PBMCs with vitiligo is increased.^25^ Furthermore, the study found that F2RL1 may be a new driver of vitiligo, closely related to reactive oxygen species, and contributes to the activation and/or migration of melanocyte‐specific CD8 + T cells. Our research found that F2RL1 as the common target gene of hsa‐miR‐340‐5p and hsa‐miR‐20a‐5p. Consequently, we speculate that hsa‐miR‐340‐5p/hsa‐miR‐20a‐5p could jointly modulate the expression of F2RL1, thereby affecting the development of vitiligo. Studies have shown that the transcriptional regulatory network constructed by the MITF gene plays a vital role in the development, differentiation, and functioning of melanocytes.^26^ This shows that it is closely related to pigment disorders. Our bioinformatic prediction showed that MITF was the target gene of hsa‐miR‐340‐5p. Hence, we hypothesize that hsa‐miR‐340‐5p/MITF may regulate the development, differentiation, and function of melanocytes. Research has shown that IL‐22 is closely related to active vitiligo, which may cause inflammation and lead to the destruction of melanocytes.^27^, ^28^ Our research indicates that IL‐22 is a common target gene of hsa‐miR‐340‐5p and hsa‐miR‐335‐5p. Therefore, we speculate that hsa‐miR‐340‐5p and hsa‐miR‐335‐5p may jointly regulate the expression of IL‐22 and cause vitiligo inflammation and melanocyte destruction. Our results disclose that a large number of vitiligo susceptibility genes (IKZF4,^29^ STRN3,^30^ ZMIZ1,^31^ CDH1^32^) were the target genes for hsa‐miR‐20a‐5p, hsa‐miR‐335‐5p, and hsa‐miR‐340‐5p. Thus, hsa‐miR‐20a‐5p, hsa‐miR‐335‐5p, and hsa‐miR‐340‐5p could serve a pivotal function in the pathogenesis of vitiligo.

In conclusion, the present work provided a profile of miRNA expression profiles in PBMCs in patients with NSV. Moreover, this study found that miRNAs are involved in vitiligo activity. We demonstrate that hsa‐miR‐20a‐5p has the potential to become a biomarker for stage assessment of NSV. Further studies should focus on investigating the exact mechanism of miRNA‐mRNA network in NSV in a large sample.

## ETHICAL APPROVAL

The study was approved by the Ethics Committee of The Second Affiliated Hospital of Nanchang University in agreement with the Declaration of Helsinki. Written informed consent was obtained from the guardians of the study subject.

## Data Availability

All relevant data are included in the manuscript. The datasets used and/or analyzed during the current study are available from the corresponding author upon request.
